# A mixed methods exploratory study tackling smoking during pregnancy in an urban Aboriginal and Torres Strait Islander primary health care service

**DOI:** 10.1186/s12889-019-6660-1

**Published:** 2019-03-25

**Authors:** Deborah A. Askew, Jillian Guy, Vivian Lyall, Sonya Egert, Lynne Rogers, Leigh-anne Pokino, Peggy Manton-Williams, Philip J. Schluter

**Affiliations:** 1 School of Clinical Medicine, Primary Care Clinical Unit, The University of Queensland, Royal Brisbane & Women’s Hospital, Level 8 Health Sciences Building, Building 16/910, Brisbane, QLD 4029 Australia; 2 Southern Queensland Centre of Excellence in Aboriginal and Torres Strait Islander Primary Health Care, Queensland Health, PO Box 52, Inala, QLD 4077 Australia; 3 School of Health Sciences, University of Canterbury – Te Whare Wānanga o Waitaha, Private Bag 4800, Christchurch, 8140 New Zealand

**Keywords:** Indigenous health, Smoking and tobacco, Health promotion, Social determinants of health

## Abstract

**Background:**

Pregnancy can be a time of joy and a time of significant stress. For many Aboriginal and Torres Strait Islander (hereafter, respectfully, Indigenous) women, cigarette smoking, even during pregnancy, is a socially sanctioned behavioural response to stress. Indigenous women smoke during pregnancy at higher rates than their non-Indigenous counterparts.

**Methods:**

A mixed methods, exploratory study, undertaken in an urban, Indigenous primary health care service, tested the impact and acceptability of a smoking cessation intervention for women pregnant with an Indigenous baby, their significant other (SO), and their primary health care service. The intervention included case management, incentivised smoking cessation support and culturally-based art activities.

**Results:**

Thirty-one pregnant women and 16 SOs participated. Nearly half attempted to quit at least once during the study, 36% (4/11) of pregnant women had quit at the 3 month assessment and two remained smoke free 1 month postpartum. Most participants self-reported a reduction in tobacco smoking. Exhaled CO confirmed this for SOs (mean reduction − 2.2 ppm/assessment wave, 95% CI: -4.0, − 0.4 ppm/assessment wave, *p* = 0.015) but not for pregnant women. Many participants experienced social and economic vulnerabilities, including housing and financial insecurity and physical safety concerns.

**Conclusions:**

Tobacco smoking is normalised and socially sanctioned in Indigenous communities and smoking is frequently a response to the multitude of stressors and challenges that Indigenous people experience on a daily basis. Smoking cessation interventions for pregnant Indigenous women must be cognisant of the realities of their private lives where the smoking occurs, in addition to the impact of the broader societal context. Narrow definitions of success focussing only on smoking cessation ignore the psychological benefit of empowering women and facilitating positive changes in smoking behaviours. Our smoking cessation intervention supported pregnant women and their SOs to manage these stressors and challenges, thereby enabling them to develop a solid foundation from which they could address their smoking. A broad definition of success in this space is required: one that celebrates positive smoking behaviour changes in addition to cessation.

## Background

Pregnancy can be a time of joy, but it can also be a time of significant stress. Many women turn to their mothers, aunties and grandmothers for support and advice at this time. However, for many Aboriginal and Torres Strait Islander (hereafter, respectfully, Indigenous) women, intergenerational sharing of knowledge and wisdom has been disrupted owing to the premature deaths of many of the older generations, and the ongoing state sanctioned destruction of families by the removal of children [1, 2]. Additionally, ongoing colonisation, discrimination, and the socioeconomic inequities in education, employment, health, income and housing, can result in Indigenous women experiencing very high levels of stress throughout their lives [3, 4]. For many, cigarette smoking, even during pregnancy, is socially sanctioned by the Indigenous community as an appropriate behavioural response to these stressors [5].

Smoking during pregnancy is one of the most important preventable risk factors contributing to the unacceptable disparity in Indigenous maternal and infant health outcomes [6]. In Australia, an estimated 43% of Indigenous women smoked during pregnancy in 2016; a notable decrease from 50% in 2009, but still significantly higher than 12% observed in non-Indigenous Australians [7]. An individual behaviourist and biomedical view of health would lay the blame squarely at the feet of the individual women, but this has three notable failings. First, the focus on individuals’ behaviours risks stigmatising women who smoke during pregnancy as non-caring, non-compliant, and dysfunctional [8]. Second, it fails to acknowledge the normalisation of smoking in Indigenous communities and the concomitant role of smoking in the maintenance of social networks and affirmation of cultural identity [5, 8, 9]. Third, it ignores the inextricable link between Indigenous people’s use of tobacco and Australia’s history of colonisation [10].

The social determinants of health paradigm is an alternate view. Here, health inequalities reflect pervasive inequalities in society, and lifestyle behaviours such as smoking are recognised as symptoms of these inequalities [11]. Therefore, the high rates of smoking among pregnant Indigenous women can be understood as a consequence of the socio-economic and socio-cultural disadvantage that Indigenous people experience in Australian society, rather than as an indicator of personal or individualised deviance or dysfunction [12].

Many Indigenous women are motivated to quit smoking during pregnancy, but stressful life events and a lack of relevant smoking cessation support are barriers to cessation [5, 8, 13, 14]. Interventions to change smoking behaviours must therefore be cognisant of each woman’s social context, address the cause and/or effect of stressors, provide evidence based smoking cessation support, and recognise the centrality of culture for her health and wellbeing [13, 15, 16]. Based on these understandings, we developed and implemented the Empowering Strong Families 4077 (ESF) smoking cessation program. The project’s Women’s Advisory Group advised that the program’s name should be strengths-based, empowering, inclusive of the family, action-oriented, and be specific for the Inala community where the study was located (4077 is Inala’s postcode, and has considerable currency in the Inala Indigenous community). Our study aimed to determine the impact of the ESF smoking cessation intervention on smoking rates of pregnant women and their significant others (SOs), and determine its feasibility and acceptability to study participants and their primary health care service.

## Methods

### Design and study setting

This exploratory, mixed methods study was conducted at the Southern Queensland Centre of Excellence in Aboriginal and Torres Strait Islander Primary Health Care (COE), a Queensland Government funded comprehensive primary health care service located in Inala, a south-western suburb of Brisbane [17]. At 5.5% (810/14,896), the percentage of the Inala population who identify as Indigenous is nearly twice that of the national percentage of 2.8%, as determined in the 2016 Australian Census [18]. The COE has approximately 10,000 patients; about 85% of whom are Indigenous [17]. Approximately 80 patients give birth each year, with approximately 50% of these being aged 25 years or younger [19].

### Participants

Women pregnant with an Indigenous baby (defined as either the mother or father self-identifying as Indigenous) of any gestational age, aged 14 to 30 years, and who smoked tobacco (or living in a smoking environment) were eligible to participate in ESF. Recognising the influence of SOs on maternal smoking [20], women were encouraged to nominate a SO to also participate. Additional eligibility criteria included being a COE patient, or willing to become one; living within a 1 hour driving radius of the COE; ability to understand written and spoken English as study materials were only available in English; and ability to provide informed consent.

Exclusion criteria included significant neurological/ cognitive impairment or incarceration at the time of recruitment. Participants who permanently moved outside of the study’s geographic catchment, suffered a miscarriage, or were incarcerated with a custodial period that extended beyond their expected time with the study were withdrawn. Additionally, SOs could be withdrawn at the request of the pregnant woman if the relationship broke down. Data collected from withdrawn participants were included unless requested otherwise.

### Recruitment

Recruitment occurred from mid-November 2016 until late-December 2017. Potential participants were identified by COE staff (general practitioners (GPs), Aboriginal Health Workers, nurses, allied health professionals), self-referral, or nomination by existing participants. ESF case managers discussed the study with each potential participant and confirmed eligibility. Written informed consent was obtained prior to data collection. With permission, ineligible patients or those declining to participate were referred to the COE Tobacco Treatment Specialist for smoking cessation support.

### The ESF intervention

Reflecting best practice in Indigenous smoking cessation programs [21, 22], our holistic multifaceted intervention aimed to support smoking cessation and/or reduction by enhancing participants’ personal agency and empowering them to address key stressors in their lives. The intervention had three key components: art activities; case management support; and incentivised smoking cessation support.

### Art activities

The art activities celebrated culture, aimed to enhance social and emotional well-being and strengthen the bonding between mother and unborn child [23–25]. Activities included pregnancy, family and newborn photographs, belly plaster-casts, pregnancy belly painting, and plaster-casts of the new baby’s hands and feet. Where possible, the artists were Indigenous and from the local community, thereby aiming to strengthen participants’ cultural and social connectivity [26].

### Case management support

The case managers provided individualised support to participants from recruitment until 3 months postpartum. Contact between case managers and participants was not limited to assessment points, but rather case managers provided support and assistance as needed and where participants felt most comfortable, for example at their homes. ESF was not a crisis support program, nor did the case managers provide 24 h, 7 day a week coverage. Therefore, participants were provided contact details of appropriate services to access in case of emergencies or crises.

### Incentivised smoking cessation support

The case managers used individualised, strengths-based, motivational interviewing strategies to encourage and enable participants to reduce or quit smoking. Free nicotine replacement therapy (NRT) was provided, using best practice guidelines for its use in pregnancy [27]. The level of exhaled carbon monoxide (CO) was assessed using Micro+ Smokerlyzer® CO monitors. This validated measure is sensitive to changes in smoking over time [28], and reductions in Smokerlyzer® monitor readings were a motivational aid for participants. These readings were also used to determine eligibility for financial incentives for smoking cessation or reduction. Shopping vouchers up to AU$190 were available for the pregnant women: AU$20 at 2 weeks post baseline if quit or reduced; AU$30 at 1 month if quit or reduced; AU$60 at 3 months if quit or AU$25 if reduced; and AU$80 1 month postpartum if quit or AU$25 if reduced. SOs could receive shopping vouchers up to AU$130: AU$20 at 2 weeks if quit or reduced; AU$30 at 1 month if quit or reduced; AU$40 at 3 months and at 1 month postpartum if quit; and AU$25 if reduced at these assessment points.

### Data collection and analysis

All participants completed assessments at baseline, 2 weeks and 1 month. Pregnant women and SOs who entered the study before 28 weeks gestation completed a 3 month assessment. A 1 month postpartum assessment was completed by those who had reached this milestone within the study data collection timeframe. Table 1 presents the schedule and broad content of assessments.

**Table 1:** Schedule and content of assessments for ESF participants (pregnant women (PW) and significant others (SOs))

Study Contact point	T0		T1		T2		T3		T4	
Timing	Baseline		2 weeks		4 weeks		12 weeks^a^		1 month postpartum^b^	
	PW	SOs	PW	SOs	PW	SOs	PW	SOs	PW	SOs
Sociodemographics	x	x								
Smoking history	x	x								
Smoking behaviours	x	x	x	x	x	x	x	x	x	x
Quit attempts	x	x	x	x	x	x	x	x	x	x
Carbon monoxide	x	x	x	x	x	x	x	x	x	x
Hooked on Nicotine Checklist (HONC)	x	x			x	x			x	x
Depression (aPHQ-9)	x	x			x	x			x	X

^a^participants entering the study late in gestation (28 weeks or more) who had birthed their babies before the three month assessment did not complete this assessment

^b^The one month postpartum assessment was completed by those who had reached this milestone within the study data collection timeframe

The baseline assessment included: self-identified ethnicity; self-reported date of birth, employment status and highest level of education; smoking history including age of, and reasons for, smoking initiation; smoking behaviours included quit attempts, time to first cigarette after waking [29], number of cigarettes per day and smoking of tobacco alternatives including illicit substances. Pregnant women were asked about smoking behaviours, including quit attempts, during previous pregnancies (if applicable). Depression was assessed using the culturally appropriate adapted 9-item Patient Health Questionnaire (aPHQ-9) [30].

For participants who reported being a current smoker at baseline, rates of smoking cessation or reduction were determined at each assessment point as were changes in participants’ smoking behaviours, personal agency for smoking cessation or reduction, and emotional wellbeing (depression).

Feasibility and acceptability of the ESF model of smoking cessation support was assessed by calculating recruitment and withdrawal rates, and participants’ engagement with the various components of the ESF intervention. Semi-structured interviews to gauge interviewees’ experiences and satisfaction with the ESF intervention were conducted with participants (seven pregnant women and six SOs) and four COE staff (two GPs, the Tobacco Treatment Specialist and the psychologist) in the 6 months from April until September 2017.

The STROBE guidelines for cross-sectional studies [31] informed reporting of quantitative results. Descriptive statistics, including means, standard deviations (SDs), frequencies and percentages were used to present participants’ baseline characteristics. Ignoring participant matching, group baseline differences between the pregnant women and SOs were assessed using Fisher’s exact test. Mean changes over time in exhaled CO and Hooked on Nicotine Checklist (HONC) score [32] were evaluated using normal generalized estimating equation (GEE) models with exchangeable correlation matrix and robust Huber-White sandwich variance estimators. Given the varying gestational age of participants at enrolment, time was indicated by assessment wave in these GEE analyses. A similar approach was employed to evaluate the change in depression indications over time, except binomial GEE models were employed. For all statistical tests, α = 0.05 was used to define significance.

A phenomenological methodology [33, 34] guided the qualitative data collection and analysis. With permission, interviews were audio-recorded and transcribed, with identifying information removed. Transcripts were analysed thematically [35] by non-Indigenous research officers. The preliminary analyses were discussed at study team workshops to ensure incorporation of Indigenous knowledge and perspectives.

### Aboriginal and Torres Strait islander community approval

We employed the National Health and Medical Research Council guidelines for ethical conduct in research with Aboriginal and Torres Strait Islander Peoples and communities [36] to develop this research. The Inala Community Jury for Aboriginal and Torres Strait Islander Health Research (a group of Aboriginal and Torres Strait Islander people from the Inala community who guide all research conducted by or through the COE) provided community approval for this research to proceed [37]. At key points throughout the study, updates were provided to the Inala Aboriginal and Torres Strait Islander community via the Community Jury and at COE staff forums.

## Results

### Participants

Forty seven people were recruited to our study: 31 pregnant women and 16 SOs. Seven pregnant women who were referred did not participate. Two referred women were ineligible: one relocated out of the study catchment and the other was incarcerated between referral and recruitment. The remaining five were eligible. Two agreed to participate, but one miscarried and the other became uncontactable prior to completion of the baseline assessment. Three declined to participate: two were not interested and one felt she was receiving sufficient support from the COE social worker. Sixteen women nominated SOs. Of these, 13 were the unborn child’s father, two were the pregnant women’s sister, and one was the woman’s sister-in-law.

### Baseline characteristics

Table 2 presents participants’ baseline characteristics. The majority of participants were Aboriginal, current smokers, and aged from 21 to 25 years. The mean age of the pregnant women was 22 years (SD 3.4 years), and 24 years (SD 7.9 years) for the SOs. Depression was observed in 29% of pregnant women and 44% of SOs (*p* = 0.35). For those answering this question, marijuana was smoked by 90% of SOs and 29% of pregnant women (Fisher’s exact test, *p* = 0.013). Amongst those smoking tobacco at baseline (Table 3), pregnant women and SOs exhibited the same level of nicotine addiction assessed through their HONC scores [32]. Ten or more cigarettes were smoked each day by 58% of SOs and 32% of pregnant women (Fisher’s exact test, *p* = 0.16). More than half of all participants (61% of pregnant women and 58% of SOs) had previously attempted to quit smoking.

**Table 2:** Baseline characteristics of pregnant women (*n* = 31) and significant others (*n* = 16) participating in the Empowering Strong Families 4077 study

	Pregnant women		Significant Other	
	n	(%)	n	(%)
*Ethnicity*				
Aboriginal	23	(74)	13	(81)
Torres Strait Islander	0	(0)	0	(0)
Aboriginal and Torres Strait Islander	2	(6)	0	(0)
Neither Aboriginal nor Torres Strait Islander	6	(19)	3	(19)
*Age (years)*				
≤ 20	8	(26)	5	(31)
21–25	17	(55)	8	(50)
26–30	6	(19)	1	(6)
≥ 31			2	(13)
*Employment status*				
Employed	8	(26)	3	(20)
Unemployed	14	(45)	9	(60)
Fulltime carer	9	(29)	3	(20)
*Highest level of education*				
Did not complete secondary schooling	17	(55)	11	(73)
Completed final year of secondary schooling	8	(26)	3	(20)
Completed post-secondary qualification	6	(19)	1	(7)
*Tobacco smoking status*				
Current smoker	23	(74)	13	(81)
Ex-smoker	6	(19)	0	(0)
Never smoked	2	(6)	3	(19)
*Marijuana smoking status*				
Yes	8	(29)	9	(90)
No	20	(71)	1	(10)
*Age of smoking initiation (years), mean (SD)*	14	(2.6)	12	(3.2)
*Previous quit attempts*				
Yes	17	(61)	7	(58)
No	11	(39)	5	(42)

**Table 3:** Changes in smoking rates, exhaled carbon monoxide levels, level of nicotine dependency, and smoking behaviours of the pregnant women and their significant others who were current smokers at baseline

	Baseline		2 weeks		1 month		3 months		1 month postpartum	
	n/N	%	n/N	(%)	n/N	(%)	n/N	(%)	n/N	(%)
*Pregnant Women*	(*N* = 23)		(*N* = 13)		(*N* = 19)		(*N* = 11)		(*N* = 13)	
* Self-reported reduction or cessation*										
Reduction	n/a	n/a	13/13	(100)	18/19	(95)	8/10	(80)	10/12	(83)
Quit attempt	n/a	n/a	5/13	(38)	8/18	(44)	7/11	(64)	4/13	(31)
Quit	n/a	n/a	0/13	(0)	0/18	(0)	4/11	(36)	2/13	(15)
Reduction and/or quit	n/a	n/a	13/13	(100)	18/19	(95)	9/11	(82)	10/13	(77)
* Time from waking until 1st cigarette (minutes)*										
< 5	4/22	(18)	2/13	(15)	3/18	(17)	0/6	(0)	2/11	(18)
5–30	8/22	(36)	3/13	(23)	1/18	(6)	0/6	(0)	3/11	(27)
31–60	1/22	(5)	4/13	(31)	5/18	(28)	4/6	(67)	1/11	(9)
> 60	9/22	(41)	4/13	(31)	9/18	(50)	2/6	(33)	5/11	(45)
*Number of cigarettes smoked each day*										
≤ 10	15/22	(68)	12/13	(92)	14/16	(88)	7/7	(100)	9/12	(75)
11–20	5/22	(23)	0/13	(0)	1/16	(6)	0/7	(0)	2/12	(17)
21–30	2/22	(9)	1/13	(8)	1/16	(6)	0/7	(0)	1/12	(8)
≥ 31	0/22	(0)	0/13	(0)	0/16	(0)	0/7	(0)	0/12	(0)
*Exhaled COppm, mean (SD)*	12.4	(5.0)	16.2	(10.6)	16.4	(12.2)	10.7	(12.8)	12.1	(9.8)
*HONC score, mean (SD)*	6.1	(2.7)	n/a	n/a	4.9	(2.6)	n/a	n/a	5.4	(3.3)
*Significant Others*	(*N* = 13)		(*N* = 7)		(*N* = 7)		(*N* = 5)		(*N* = 5)	
*Self-reported reduction or cessation*										
Reduction	n/a	n/a	6/7	(86)	7/7	(100)	4/4	(100)	4/5	(80)
Quit Attempt	n/a	n/a	2/7	(29)	3/7	(43)	3/5	(60)	3/5	(60)
Quit	n/a	n/a	0/7	(0)	0/7	(0)	1/5	(20)	0/4	(0)
Reduction and/or quit	n/a	n/a	6/7	(86)	7/7	(100)	5/5	(100)	4/5	(80)
*Time from waking until 1st cigarette (minutes)*										
< 5	5/12	(42)	3/7	(43)	0/7	(0)	0/4	(0)	1/5	(20)
5–30	3/12	(25)	1/7	(14)	3/7	(43)	2/4	(50)	0/5	(0)
31–60	3/12	(25)	1/7	(14)	2/7	(29)	0/4	(0)	2/5	(40)
> 60	1/12	(8)	2/7	(29)	2/7	(29)	2/4	(50)	2/5	(40)
*Number of cigarettes smoked each day*										
≤ 10	5/12	(42)	5/7	(71)	5/6	(83)	3/5	(60)	4/5	(80)
11–20	3/12	(25)	0/7	(0)	0/6	(0)	0/5	(0)	0/5	(0)
21–30	3/12	(25)	1/7	(14)	0/6	(0)	1/5	(20)	0/5	(0)
≥ 31	1/12	(8)	1/7	(14)	1/6	(17)	1/5	(20)	1/5	(20)
*Exhaled COppm, mean (SD)*	21.4	(13.3)	27.3	(19.8)	12.7	(9.2)	14.2	(9.2)	16.8	(6.8)
*HONC score, mean (SD)*	6.1	(2.9)	n/a	n/a	5.7	(3.2)	n/a	n/a	7.8	(1.8)

*COppm* Carbon monoxide part per million, *HONC* Hooked on Nicotine Checklist, *n/a* Not available

The majority of participants lived in households where at least one person smoked. Among pregnant women, the median household size was four occupants (range: 2 to 11 occupants), with a median of two smokers per household (range: 0 to 6 smokers). The household where nobody smoked, the woman’s partner, a smoker, lived elsewhere.

The mean gestational age at recruitment was 17 weeks (range: 4 to 35 weeks). Fourteen women (45%) were pregnant with their first baby, 35% (*n* = 11) with their second, and the remaining 19% (*n* = 6) of women already had two or more babies. All 12 of the women who were smokers and multiparous had smoked during their previous pregnancies.

**Fig. 1:**
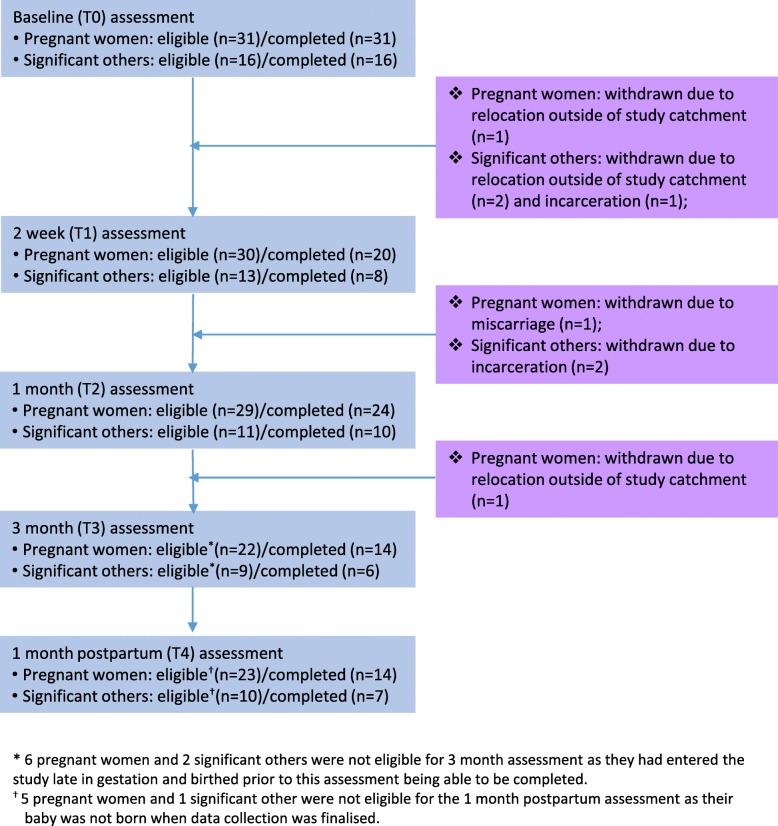
ESF study participant flow

Three pregnant women and five SOs withdrew from the study: one pregnant woman miscarried; two pregnant women and two SOs moved out of the study catchment; and three SOs were incarcerated (Fig. 1). However, with the exception of the T2 assessment which was completed by 83% (24/29) of pregnant women and 91% (10/11) of SOs, data capture rates were low. The T1 assessment was completed by 67% (20/30) of pregnant women, 64% (14/22) completed the T3 assessment, and 61% (14/23) completed the T4 assessment at 1 month postpartum. Similar rates were observed for the SOs: 62% (8/13) completed the T1 assessment, 67% (6/9) completed the T3 assessment, and 70% (7/10) completed the T4 assessment (Fig. 1).

### Engagement and satisfaction with the program

Participants and health professionals were all highly satisfied with the ESF intervention. In particular, they valued that the approach was flexible, strength-based, family centred, holistic, relationship-based, and participant-led.

*“They were willing to work with me and not pressure me about giving up, you can just slowdown in time and I thought that was good, yeah. Because a lot of people just expect you to quit straight up, not just like want to work with you … but we just talked a lot and, just little things that, to avoid smoking and over time I have cut down heaps” (pregnant woman).*

These features of value facilitated participants’ engagement, enabled the delivery of individualised smoking cessation support, and contributed to the confidence that health professionals had in the program with one GP commenting ….

*“it’s making people feel good about themselves, so a healing activity that’s different to just our biomedical or our psychological model of sitting down counselling someone, and that’s different”(GP).*

In the 20 months from commencement of recruitment to study end in June 2018, there was an average of 12.2 contacts between pregnant women and case managers (range: 2 to 34 contacts) and 6.4 contacts between SOs and case managers (range: 1 to 18). In addition to the scheduled assessments, the purposes of the contacts included supporting participants to access medical, mental health, antenatal and dental care; advocating with governmental agencies for participants experiencing housing and/or financial insecurity; facilitating access to emergency food relief for participants experiencing food insecurity; supporting participants experiencing domestic violence to access safe refuge; supporting participants meet obligations set by the criminal justice system; and being a ‘safe harbour’ for participants as their experienced the vicissitudes of life. Despite our participants being COE patients, they typically had not shared with COE health professionals about the complexity or the multiplicity of challenges they were experiencing. For example, a woman could have a history of childhood sexual abuse and substance abuse, her unborn child’s father could be incarcerated owing to domestic and sexual violence towards her, she could be experiencing financial and housing insecurity, she may have mental health problems, and she may prematurely birth her child. The greater the complexity of participants’ lives, the greater their need for support and therefore, the more contact with their case manager.

Smoking cessation advice and support was available to participants throughout their involvement with ESF, and NRT was used by 13 pregnant women (gum, patches, spray, inhaler or lozenges), and one was given NRT but did not use it. Six SOs used NRT. Four pregnant women and one SO considered NRT to be the most important support in assisting them to reduce the number of cigarettes smoked each day and attempting to quit.

A total of 11 women engaged with at least one art activity, with another four wanting to but circumstances such as the early arrival of the baby prevented this occurring. Five women had their belly painted, six had pregnancy photos taken, five had newborn and/or family photographs, and two had plaster casts made of their babies’ hands and feet. Positive feedback was received from all women who participated in the arts program, as articulated by this pregnant woman…

*[the arts activities made me feel]… “more in love with my baby, yep. Oh, it made me feel so good. Yeah. I never felt so good in all three pregnancies … I was so glowing and everything with my third, with [child’s name] and in just doing this program.” pregnant woman*

### Smoking status

None of the non- or ex-smokers took up smoking during the study, including those who had quit after becoming pregnant and before entering the study. Of the 23 pregnant women smoking at baseline, the majority self-reported tobacco smoke reduction at each assessment (Table 3). Quit attempts were reported by 38% (5/13) at 2 weeks, 44% (8/18) at 1 month, 64% (7/11) at 3 months and 31% at 1 month postpartum. Four women (36%) had quit at the 3 month assessment, with two (15%) remaining smoke free at 1 month postpartum. However, there was no significant decrease in exhaled CO (GEE, *p* = 0.53) or HONC scores [32] (GEE, *p* = 0.31) over the study duration (Table 3).

Quit attempts were reported by 29% of SOs (2/7) at 2 weeks, 43% (3/7) at 1 month, and 60% (3/5) at both the 3 months post-baseline and 1 month postpartum assessments. One SO had a successful quit attempt at 3 months. At each assessment, the majority of the 13 SOs smoking at baseline reported reducing the amount they smoked (Table 3). Exhaled CO decreased amongst these SOs over the study duration (GEE mean reduction − 2.2 ppm/assessment wave, 95% CI: -4.0, − 0.4 ppm/assessment wave, *p* = 0.015) but not for HONC scores [32] (GEE, *p* = 0.15) (Table 3).

### Mental health

Amongst the pregnant women, 29% (9/31) were depressed at baseline; 24% (5/21) at 1 month; and 29% (4/14) at 1 month postpartum (GEE, *p* = 0.89). Amongst SOs, 44% (7/16) were depressed at baseline; 30% (3/10) at 1 month; and 29% (2/7) at 1 month postpartum (GEE, *p* = 0.57).

## Discussion

The ESF smoking cessation intervention was predicated on the understanding that the high rates of smoking among Indigenous people are a sequela of their differential access to power and resources, and their lived experiences of racism, oppression and poverty. For our participants, these factors manifested as housing, food and financial insecurity, a lack of safety and security, and histories of complex trauma. Nevertheless, nearly all participants reported making positive changes to their smoking behaviours by reducing the number of cigarettes they smoked each day or attempting to quit, although this was not reflected in reductions in exhaled CO for the pregnant women. Four pregnant women quit during their pregnancy (36%) and two remained smoke free in the stressful early postpartum period. In comparison, a systematic review of studies aiming to reduce smoking during pregnancy found no clear effect of any psychosocial interventions among Indigenous women [21]. SOs reduced the amount they smoked, evidenced by a reduction in exhaled CO. Participants and health service staff reported high levels of satisfaction with the program. Participants reported developing trusting relationships with the case managers through which they felt safe to disclose the realities of their lives, they felt supported, and some reported developing more positive outlooks on life, improved bonding with their unborn child, and a shifting identity from smoker to non-smoker. Health service staff appreciated the non-biomedical wrap-around support provided by ESF for patients they knew were experiencing disadvantages.

Our study had some important strengths, particularly the Indigenous leadership and community input into the development and implementation of the model of care. The model of care was flexible, and focussed on meeting each individual’s needs, and accounted for the social determinants of health including historical antecedents, community norms and cultural strengths. Additionally, the ESF model of care was fully embedded within the primary health care service, ensuring continuous, comprehensive and coordinated care of participants. For example, participants with depression were supported by their GP and the COE mental health care team, in addition to activities coordinated by the Aboriginal Health Workers, for example men’s and women’s groups. The exploratory study design enabled us to adapt the model of care as we gained more understanding about the context of our intervention, and particularly, the reality of participants’ lives. However, these adjustments limit our capacity to draw generalisable conclusions about the model’s efficacy.

Further limiting the generalisability of our results was the small sample size and the incomplete data capture. The multitude and complexity of challenges experienced by participants required case managers to invest considerable time in supporting participants, thus limiting the number of participants that could be recruited at any one time. The project’s timeframe further limited the potential sample size. Funding was allocated in September 2016, recruitment commenced in November 2016, and due to the non-negotiable end date of 30 June 2018, recruitment ended in December 2017. A longer recruitment period would likely have increased participation. Two factors could explain the incomplete data capture. Firstly, many of our participants were experiencing a multitude of stressful events, often simultaneously, and therefore addressing these stressors would take precedence over completion of a scheduled assessment. Second, some participants were unable to be contacted when assessments were due, potentially due to a lack of phone credit, temporary relocation, or becoming socially isolated due to, for example, mental health or domestic violence.

With the intensive support provided to ESF participants, over one third quit smoking at 3 months, and two participants were still smoke free 1 month postpartum. These results, while promising in comparison to other published studies [21], do not negate the fact that the majority of participants continued to smoke. Also highly relevant is the role of tobacco in Indigenous communities as a mechanism for affirming cultural identity and strengthening community social capital [8, 9]. The very act of sharing a cigarette for many Indigenous people has deep symbolism. It maintains cultural connectivity and interpersonal relationships through reciprocity and sharing of resources [9] and creates a perceived ‘safe haven’ and socially acceptable and accessible antidote to the lived realities of oppression, racism and poverty [8]. The reinforcement of identity will likely always take precedence over the imperative of the neoliberal ideology and definition of a responsible citizenship [38]. Therefore, narrow definitions of success such as complete abstinence may need to be reconsidered in this space as a reduction in smoking can be celebrated as a psychologically empowering achievement for participants [5] and potentially paving the way for future smoking cessation.

## Conclusions

ESF 4077 was a small study in one primary health care service that achieved some positive results with four women quitting in late pregnancy, two remaining smoke free 1 month postpartum, and nearly 100% of women and SOs making positive changes in their smoking. Much was learnt from this research about the challenges faced by pregnant urban Indigenous women, and therefore the support required to enable and empower them to contemplate and embark on smoking cessation. We had anticipated that participants would be experiencing stress that would limit their capacity to quit smoking, we had not anticipated that participants would be experiencing multiple stressors and trauma simultaneously, which overlay the historical and intergenerational traumas experienced by Indigenous people rendering smoking cessation as a lessor priority. Further research is needed to identify if our participants’ were empowered to better manage the vicissitudes and challenges of life, if ESF participants’ babies had a better start in life than they would have otherwise, and if the positive changes to smoking behaviours observed during ESF ultimately led to cessation. Furthermore, we are continuing to analyse our data to identify our model of care’s key features of value to enable scaling up and implementation in other locations. Ideally, and subject to funding, the model of care will be tested in a randomised controlled trial to determine its effectiveness. While the health harms associated with maternal smoking during pregnancy are unquestionable, more research is needed to understand both the realities of everyday life in which this smoking occurs [8] and the impact of the broader societal context, and how best to support urban Indigenous women to live full and rewarding lives, including taking control of their smoking stories.

## Abbreviations

aPHQ-9: Adapted Patient Health Questionnaire-9 item
CI: Confidence Interval
COE: Southern Queensland Centre of Excellence in Aboriginal and Torres Strait Islander Primary Health Care
COppm: Carbon monoxide parts per million
ESF: Empowering Strong Families 4077
GEE: Generalised Estimating Eqs.
GP: General Practitioner
HONC: Hooked on Nicotine Checklist
NRT: Nicotine Replacement Therapy
PW: Pregnant women
SO: Significant Other

## References

[1] Heath F, Bor W, Thompson J, Cox L (2011). Diversity, disruption, continuity: parenting and social and emotional wellbeing amongst aboriginal peoples and Torres Strait islanders. Aust N Z J Fam Ther.

[2] Kilcullen M, Swinbourne A, Cadet-James Y, McCoy B, Stewart P, Poroch N (2012). Factors affecting resilience of Aboriginal and Torres Strait Islander grandmothers raising their grandchildren. Urban health: Strengthening our voice, culture and partnerships.

[3] Heath DL, Panaretto K, Manessis V, Larkins S, Malouf P, Reilly E (2006). Factors to consider in smoking interventions for indigenous women. Aust J Prim Health.

[4] Cosh S, Hawkins K, Skaczkowski G, Copley D, Bowden J (2015). Tobacco use among urban aboriginal Australian young people: a qualitative study of reasons for smoking, barriers to cessation and motivators for smoking cessation. Aust J Prim Health..

[5] DiGiacomo M, Davidson PM, Davison J, Moore L, Abbott P (2007). Stressful life events, resources, and access: key considerations in quitting smoking at an aboriginal medical service. Aust N Z J Public Health.

[6] Mund M, Louwen F, Klingelhoefer D, Gerber A (2013). Smoking and pregnancy — a review on the first major environmental risk factor of the unborn. Int J Environ Res Public Health.

[7] Australian Institute of Health and Welfare (2018). Australia’s mothers and babies 2016 - in brief.

[8] Bond C, Brough M, Spurling GK, Hayman N (2012). ‘It had to be my choice’ indigenous smoking cessation and negotiations of risk, resistance and resilience. Health Risk Soc.

[9] Passey ME, Gale JT, Sanson-Fisher RW (2011). “It’s almost expected”: rural Australian aboriginal women's reflections on smoking initiation and maintenance: a qualitative study. BMC Womens Health.

[10] Lovett R, Thurber KA, Maddox R. The Aboriginal and Torres Strait Islander smoking epidemic: what stage are we at, and what does it mean? Public Health Res Pract. 2017;27(4):e2741733. 10.17061/phrp2741733. 10.17061/phrp2741733 29114713

[11] Wilkinson RG, Marmot M (2003). Social determinants of health: the solid facts.

[12] Marmot M (2011). Social determinants and the health of indigenous Australians. Med J Aust.

[13] Small S, Porr C, Swab M, Murray C (2018). Experiences and cessation needs of indigenous women who smoke during pregnancy: a systematic review of qualitative evidence. JBI Database System Rev Implement Rep.

[14] Wood L, France K, Hunt K, Eades S, Slack-Smith L (2008). Indigenous women and smoking during pregnancy: knowledge, cultural contexts and barriers to cessation. Soc Sci Med.

[15] Boucher J, Konkle AT. Understanding Inequalities of Maternal Smoking--Bridging the Gap with Adapted Intervention Strategies. Int J Environ Res Public Health. 2016;13(3). 10.3390/ijerph13030282. 10.3390/ijerph13030282 PMC4808945 26959037

[16] Gould GS, Cadet-James Y, Clough AR (2016). Getting over the shock: taking action on indigenous maternal smoking. Aust J Prim Health..

[17] Hayman NE, Askew DA, Spurling GK (2014). From vision to reality: a Centre of excellence for aboriginal and Torres Strait islander primary health care. Med J Aust.

[18] Australian Bureau of Statistics (2017). 2016 Census QuickStats.

[19] Maher CM, Spurling GK, Askew DA (2014). Health and well-being of urban aboriginal and Torres Strait islander women at their first antenatal visit: a cross-sectional study. Aust N Z J Obstet Gynaecol.

[20] Bauld L, Graham H, Sinclair L, Flemming K, Naughton F, Ford A (2017). Barriers to and facilitators of smoking cessation in pregnancy and following childbirth: literature review and qualitative study. Health Technol Assess.

[21] Chamberlain C, O'Mara-Eves A, Porter J, Coleman T, Perlen SM, Thomas J, et al. Psychosocial interventions for supporting women to stop smoking in pregnancy. Cochrane Database Syst Rev. 2017;2. 10.1002/14651858.CD001055.pub5. 10.1002/14651858.CD001055.pub5 PMC6472671 28196405

[22] Minichiello A, Lefkowitz ARF, Firestone M, Smylie JK, Schwartz R (2016). Effective strategies to reduce commercial tobacco use in indigenous communities globally: a systematic review. BMC Public Health.

[23] Davies CR, Knuiman M, Wright P, Rosenberg M (2014). The art of being healthy: a qualitative study to develop a thematic framework for understanding the relationship between health and the arts. BMJ Open.

[24] Archibald L, Dewar J (2010). Creative arts, culture, and healing: building an evidence base. Pimatisiwin: J Aborig Indigenous Aborig Community Health.

[25] Dyer G, Hunter E (2009). Creative recovery: art for mental health’s sake. Australas Psychiatry..

[26] Guerin P, Guerin B, Tedmanson D, Clark Y (2011). How can country, spirituality, music and arts contribute to indigenous mental health and wellbeing?. Australas Psychiatry.

[27] Zwar N, Richmond R, Borland R, Peters M, Litt J, Bell J (2014). Supporting smoking cessation: a guide for health professionals: Royal Australian College of General Practitioners.

[28] Christensen AE, Tobiassen M, Jensen TK, Wielandt H, Bakketeig L, Host A (2004). Repeated validation of parental self-reported smoking during pregnancy and infancy: a prospective cohort study of infants at high risk for allergy development. Paediatr Perinat Epidemiol.

[29] Baker TB, Piper ME, McCarthy DE, Bolt DM, Smith SS, Transdisciplinary Tobacco Use Research Center Tobacco D (2007). Time to first cigarette in the morning as an index of ability to quit smoking: implications for nicotine dependence. Nicotine Tob Res.

[30] Brown A, Mentha R, Howard M, Rowley K, Reilly R, Paquet C (2016). Men, hearts and minds: developing and piloting culturally specific psychometric tools assessing psychosocial stress and depression in central Australian aboriginal men. Soc Psychiatry Psychiatr Epidemiol.

[31] von Elm E, Altman DG, Egger M, Pocock SJ, Gotzsche PC, Vandenbroucke JP (2007). The strengthening the reporting of observational studies in epidemiology (STROBE) statement: guidelines for reporting observational studies. Epidemiology..

[32] Wellman RJ, DiFranza JR, Savageau JA, Godiwala S, Friedman K, Hazelton J (2005). Measuring adults’ loss of autonomy over nicotine use: the hooked on nicotine checklist. Nicotine Tob Res.

[33] Bevan MT (2014). A method of phenomenological interviewing. Qual Health Res.

[34] Groenewald T (2004). A phenomenological Research design illustrated. Int J Qual Methods.

[35] Braun V, Clarke V (2006). Using thematic analysis in psychology. Qual Res Psychol.

[36] National Health and Medical Research Council (2018). Ethical conduct in research with Aboriginal and Torres Strait Islander Peoples and communities: Guidelines for researchers and stakeholders.

[37] Bond C, Foley W, Askew D (2016). “It puts a human face on the researched”--a qualitative evaluation of an indigenous health research governance model. Aust N Z J Public Health.

[38] Lupton D, ProQuest (1995). The imperative of health: public health and the regulated body.

